# Differential effects of excitatory and inhibitory heterogeneity on the gain and asynchronous state of sparse cortical networks

**DOI:** 10.3389/fncom.2014.00107

**Published:** 2014-09-12

**Authors:** Jorge F. Mejias, André Longtin

**Affiliations:** ^1^Center for Neural Science, New York UniversityNew York, NY, USA; ^2^Department of Physics, University of OttawaOttawa, ON, Canada; ^3^Department of Cellular and Molecular Medicine, University of OttawaOttawa, ON, Canada

**Keywords:** heterogeneity, asynchronous state, gain control, mean-field, cortical networks, signal detection

## Abstract

Recent experimental and theoretical studies have highlighted the importance of cell-to-cell differences in the dynamics and functions of neural networks, such as in different types of neural coding or synchronization. It is still not known, however, how neural heterogeneity can affect cortical computations, or impact the dynamics of typical cortical circuits constituted of sparse excitatory and inhibitory networks. In this work, we analytically and numerically study the dynamics of a typical cortical circuit with a certain level of neural heterogeneity. Our circuit includes realistic features found in real cortical populations, such as network sparseness, excitatory, and inhibitory subpopulations of neurons, and different cell-to-cell heterogeneities for each type of population in the system. We find highly differentiated roles for heterogeneity, depending on the subpopulation in which it is found. In particular, while heterogeneity among excitatory neurons non-linearly increases the mean firing rate and linearizes the f-I curves, heterogeneity among inhibitory neurons may decrease the network activity level and induces divisive gain effects in the f-I curves of the excitatory cells, providing an effective gain control mechanism to influence information flow. In addition, we compute the conditions for stability of the network activity, finding that the synchronization onset is robust to inhibitory heterogeneity, but it shifts to lower input levels for higher excitatory heterogeneity. Finally, we provide an extension of recently reported heterogeneity-induced mechanisms for signal detection under rate coding, and we explore the validity of our findings when multiple sources of heterogeneity are present. These results allow for a detailed characterization of the role of neural heterogeneity in asynchronous cortical networks.

## 1. Introduction

Mathematical models of neurons and neural circuits have become, in the last couple of decades, a highly valuable tool to analyze and understand real neural systems, from single cell to behavior. Models are commonly used to test hypotheses or to support experimental observations, and their potential usefulness increases as their predictions are refined to account for the actual behavior of neurons (Gerstner and Naud, 2009). While it is not uncommon to see a high level of biophysical detail in single-neuron models, most of these details are usually neglected when modeling larger systems, such as neural circuits of thousands of neurons, for the sake of simplicity.

A particularly interesting case is the natural intrinsic variability found in the biophysical properties of neurons, which is averaged out in most theoretical and computational modeling studies. Real neural systems display a significant level of cell-to-cell diversity at the neuron level, even among same-class neurons, as well as other differences at the subcellular or synaptic level (Bannister and Larkman, 1995a,b; Reyes et al., 1998; Hausser and Mel, 2003; Jinno et al., 2007). Experimental observations indicate that this type of structural heterogeneity has non-trivial effects on several neural information processing mechanisms. For instance, neural heterogeneity has been shown to have an impact in burst coding *in vivo* (Avila-Akerberg et al., 2010) and in envelope coding and non-linear responsiveness of the electroreceptors of weakly electric fish (Savard et al., 2011). The presence of a certain level of heterogeneity at the cell-to-cell level has also been recently reported to have a beneficial role for population coding (Marsat and Maler, 2010; Tripathy et al., 2013), and it can also induce the decorrelation of neuronal firing and the optimization of information content (Padmanabhan and Urban, 2010; Angelo et al., 2012; Urban and Tripathy, 2012). These experimental observations can not be explained by neural circuit models where, for instance, any given pyramidal neuron is perfectly identical to all the other pyramidal neurons in the system. Models which take into account the intrinsic heterogeneity of neural systems are, therefore, necessary to understand neural coding.

In response to this increasing body of evidence, a significant number of theoretical and computational studies, especially in the last years, have contributed to explaining the properties and dynamics of networks of heterogeneous neurons. In particular, the role of heterogeneity on synchronization has been extensively studied (Golomb and Rinzel, 1993; White et al., 1998; Neltner et al., 2000; Golomb et al., 2001; Denker et al., 2004; Talathi et al., 2008, 2009; Luccioli and Politi, 2010; Olmi et al., 2010; Brette, 2012; Mejias and Longtin, 2012). More recently, the effect of neural heterogeneities on neuronal correlations (Chelaru and Dragoi, 2008; Yim et al., 2013), detection of weak signals (Tessone et al., 2006; Perez et al., 2010) and different types of neural coding (Chelaru and Dragoi, 2008; Savard et al., 2011; Mejias and Longtin, 2012; Hunsberger et al., 2014) have drawn special attention as well. Novel approaches and mean-field approximations to tackle the problem of heterogeneity have also been recently proposed (Nicola and Campbell, 2013; Yim et al., 2013). These studies, however, typically focus on one type of cell (such as pyramidal neurons) and consider the presence of heterogeneity on this specific population. The possible—and potentially relevant—interplay between populations of different cell types, each one of them presenting its own heterogeneity level, has remained a goal for future work and has not been fully addressed yet.

In this work, we analytically and numerically study the properties of a typical cortical circuit with cell-specific heterogeneity levels. Our basic circuit is constituted by a population of excitatory (i.e., pyramidal) neurons and a population of inhibitory neurons (i.e., interneurons), connected in a sparse manner. Both the excitatory and the inhibitory populations have their own independent level of intrinsic heterogeneity. This allows us to quantitatively study the effect of heterogeneity of a given population (excitatory, or inhibitory, or both) and to characterize its impact on the whole network dynamics. Our results indicate highly differentiated roles for heterogeneity, depending on the population in which it is introduced. In particular, heterogeneity among excitatory neurons (which we call here *excitatory heterogeneity*) non-linearly increases the mean firing rate of the whole network and linearizes the input/output f-I curves. On the other hand, heterogeneity among inhibitory neurons (*inhibitory heterogeneity*) may decrease the network activity level and induce divisive gain effects in the f-I curves of the excitatory population, providing an effective gain control mechanism to influence the flow of information across the network. We also compute the conditions for stability of the network activity and provide an extension of the recently described heterogeneity-induced mechanism which optimizes information transmission under rate coding (Mejias and Longtin, 2012). Our novel mean-field approach extends our previous theoretical results for fully connected excitatory neurons (Mejias and Longtin, 2012) to cortical-like sparsely connected networks of heterogeneous excitatory and inhibitory cells, providing a strong analytical tool to characterize the role of neural heterogeneity in cortical networks.

## 2. Materials and methods

**Figure 1:**
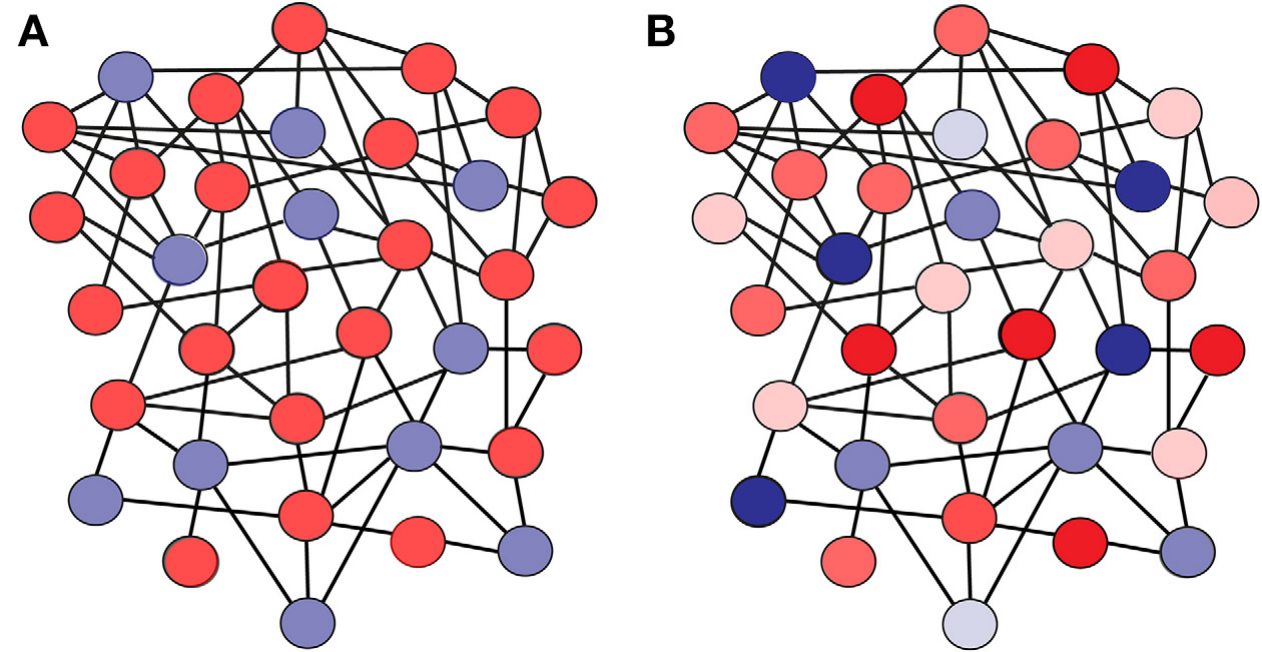
**Scheme of a network where (A) all the neurons have identical firing thresholds, and (B) each neuron in the network has a particular firing threshold value**. The color code illustrates the value of the firing threshold for each neuron (lighter color tones mean lower firing threshold, while darker tones mean higher firing thresholds). Excitatory neurons are shown in red, and inhibitory neurons in blue.

We consider a sparsely connected network of *N* integrate-and-fire neurons (see Figure 1), where any two given neurons are unidirectionally connected with a probability ϵ (the average number of synapses onto a given neuron is then *K* ≡ ϵ *N*). A subset of this population is constituted by γ *N* excitatory neurons, while the remaining (1 − γ)*N* neurons in the network are inhibitory. A given neuron *i* is governed by the dynamics

(1) $$\tau_{m}\frac{dV_{i}(t)}{dt}=-V_{i}(t)+RI_{i}^{ext}(t)+RI_{i}^{net}(t),$$

where τ*_m_* is the neuron membrane time constant, *V_i_* is the membrane potential of the *i* − *th* neuron in the network, *R* is the membrane resistance, and *I^ext^_i_*, *I^net^_i_* are the external and recurrent input to the *i* − *th* neuron, respectively. Each neuron *i* is assumed to fire an action potential (AP) every time *V_i_* reaches a certain firing threshold, and after that the membrane potential is reset to *V_r_* for a time period τ_*ref*_. The external and recurrent input to the *i* − *th* neuron are given, respectively, by

(2) $$RI_{i}^{ext}(t)=\mu_{i}+\sigma \sqrt{\tau_{m}}\xi_{i}(t),$$

(3) $$RI_{i}^{net}(t)=\tau_{m}\sum_{j}\sum_{k}J_{ij}\;\delta (t-t_{j}^{k}),$$

where μ_*i*_ is a constant input, ξ*_i_(t)* is a Gaussian white noise of zero mean and unitary variance, σ is the noise intensity, *J_ij_* is the coupling strength of the synapse from neuron *j* to neuron *i* (considered zero if there is not such a synapse between both neurons), and the *k* − *th* spike from neuron *j* arrives at neuron *i* at *t^k^_j_*. The synaptic coupling strength between two neurons *i, j* takes the value *J_ij_* = *J*_αβ_, where α = {*E, I*} is a label denoting the population to which the postsynaptic neuron belongs, and β = {*E, I*} denotes the population to which the presynaptic neuron belongs. We define the external input to the network as μ_*i*_ = μ (arriving at all excitatory neurons), and we also define a constant bias μ_*i*_ = μ_0_ for all inhibitory neurons.

In this framework, we assume that each neuron of the network is characterized by a different distance-to-threshold value, which may be related with several biophysical properties (such as the membrane resistance, the firing threshold, or extra non-linear considerations). We assume here that such heterogeneity in the distance-to-threshold corresponds to heterogeneity in firing threshold values, although heterogeneities in distance-to-threshold can be translated into heterogeneities in other kind of parameters in more sophisticated neuron models. In particular, each excitatory neuron has a firing threshold θ_*E, i*_ which is randomly distributed following a Gaussian profile *P_E_* (θ*_E_*) with mean θ and standard deviation *w_E_*. Equivalently, each inhibitory neuron has a firing threshold θ_*I, i*_ randomly chosen from a Gaussian distribution *P_I_*(θ*_I_*) with mean θ and standard deviation *w_I_* (see Figure 1). Such heterogeneity serves to reflect some of the variability in the individual excitability properties of neurons found in actual neural systems, while treating separately the heterogeneity of excitatory and inhibitory cells will allow us to discern the effects caused by each population. For convenience, we will define a *low-threshold neuron* as a neuron whose firing threshold value (or, more precisely, its distance between the threshold for spiking and its resting state in absence of input) is below the average for its population (i.e., excitatory or inhibitory). A *high-threshold neuron* will therefore have a firing threshold value which is higher than the average for its population.

In the following, and unless specified otherwise, we choose *K* = 200 connections (in simulations, ϵ = 0.2 for *N* = 1000 neurons), γ = 0.8, τ_*m*_ = 20 ms, *V_r_* = 10 mV, σ = 3 mV, θ = 20 mV, τ_*ref*_ = 5 ms, *J_EE_* = *J_IE_* = 0.05 mV, and *J_EI_* = *J_II_* = −0.08 mV. These parameter values are within the physiological range for cortical neurons, and similar values have been used in previous modeling studies (Brunel and Hakim, 1999; Brunel, 2000). When computing the response of the system (for instance, the network mean firing rate for a given heterogeneity value), we average over 10 trials (or simulation runs on a random realization of the connectivity matrix) of 10 s each. The results presented in this work hold for these other parameter choices as well.

Together with the numerical simulations of the neural network described above, we have obtained an analytical mean-field solution of the model, which is described in detail in the Supplementary Material (Section Mean-field approach). In short, we have employed the diffusion approximation in the input to a single IF neuron to compute its mean firing rate in steady state conditions (Tuckwell, 1989; Brunel, 2000). Since the input to any given neuron will depend on the activity of the whole network (due to the recurrent nature of the system), we can average over the heterogeneity and obtain a mean-field description of the excitatory and inhibitory network mean firing rate, which will be given, respectively, by ν*_E_* = Φ*_E_* (ν*_E_*, ν*_I_*, *w_E_*, *w_I_*) and ν*_I_* = Φ*_I_* (ν*_E_*, ν*_I_*, *w_E_*, *w_I_*) (see Section Mean-field approach in Supplementary Material for an explicit form of these functions). An analytical estimation of the stability of this solution has been obtained as well (see Supplementary Material). The heterogeneity parameters *w_E_* and *w_I_* have an important effect on the mean firing rates, and allow us to use this mean-field solution, together with numerical simulations of the network, to explore the properties of the system.

## 3. Results

### 3.1. Effect of heterogeneity on mean firing rate

**Figure 2:**
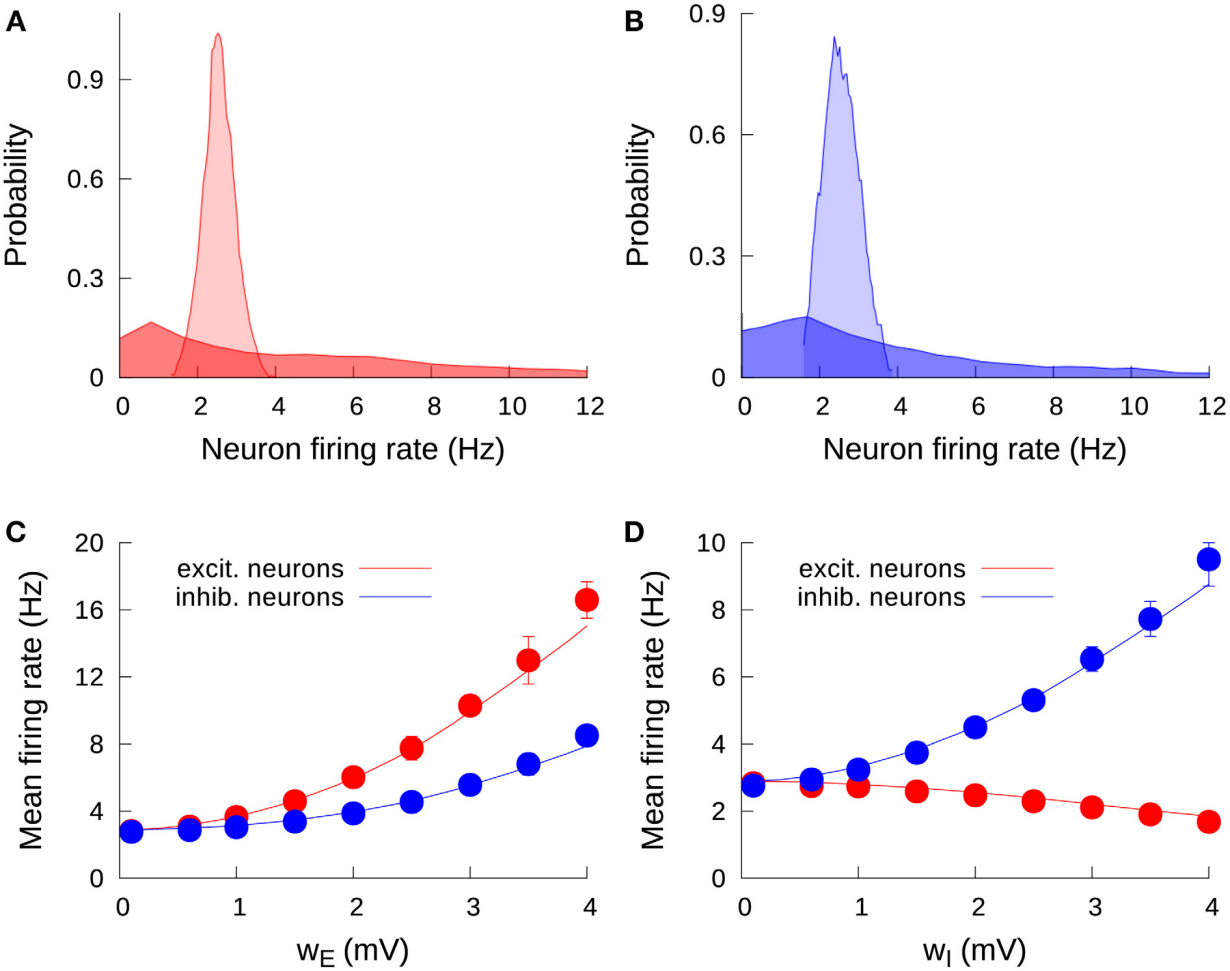
**Effect of heterogeneity on the stationary firing rates. (A)** Probability density function of individual mean firing rates of excitatory neurons, for *w_E_* = 0.1 mV (light red) and *w_E_* = 2 mV (dark red). One can observe the spread of firing rate values as a consequence of the increase in the excitatory heterogeneity. **(B)** Distribution of individual mean firing rates of inhibitory neurons, for *w_I_* = 0.1 mV (light blue) and *w_I_* = 2 mV (dark blue). **(C)** Effect of the excitatory heterogeneity on the network mean firing rate. Solid lines correspond to the mean-field solution, while symbols are the results from numerical simulations. Here and in the following, error bars (which may be within symbol size) denote standard deviation over trials. **(D)** Same as in **(C)**, but for the effect of inhibitory heterogeneity. We set a fixed value *w_I_* = 0.1 mV for **(A,C)**, and *w_E_* = 0.1 mV for **(B,D)**. For all panels, the external input is determined by μ = μ_0_ = 15 mV. Note the different scale for the vertical axis in **(C,D)**.

Our first step is to understand the effect of an increase of the level of cell-to-cell heterogeneity on the stationary firing rate of the neurons in the network. Due to input noise and the sparseness of the network (which leads to a different number of incoming connections for each neuron), the neurons in the network are not characterized by a common unique mean firing rate (even in the absence of threshold heterogeneity), but rather each neuron has an individual mean firing rate, distributed around an average value following a Gaussian-like profile. This can be seen in Figure 2A (in light red, for excitatory neurons) and Figure 2B (in light blue, for inhibitory neurons). When we consider some degree of heterogeneity in the neuron firing thresholds, this original distribution of firing rates becomes wider and spans over a large range of firing rate values. For instance, a heterogeneity level of only *w_E_* = 2 mV for excitatory neurons lead the excitatory firing rate distribution from the previous narrow, Gaussian-like profile to a broad, long-tailed distribution which contains firing rates from zero to even tens of Hertz (Figure 2A, dark red). The same effect is observed for the inhibitory population: an increase in *w_I_* from 0 to 2 mV leads from a narrow, peaked distribution (Figure 2B, light blue) to a long-tailed one (dark blue). Excitatory (inhibitory) heterogeneity has also an effect on the shape of the inhibitory (excitatory) firing rate distribution, although it is not as strong as the effect of heterogeneity in a given population on the firing rate distribution of that same population (not shown).

Heterogeneity has a significant effect not only on the shape of the distributions, but on the mean firing rate of the populations in the network as well. The case of a single excitatory population was already considered in a previous work (Mejias and Longtin, 2012), where it was shown that an increase of neural heterogeneity triggered the appearance of a group of low-threshold neurons with higher firing rates (similar to the long tail in the dark-colored distributions in Figures 2A,B), and this group produced an extra recurrent input on the high-threshold neurons forcing them to increase their firing rate. The effect had a strong collective component, since a simple firing rate increase in the low-threshold neurons would have been at least partially compensated by a decrease in the high-threshold neurons, were they not connected to each other. The overall recurrent activity generated by the low-threshold neurons contributed to avoid a sudden drop in the firing rate of high-threshold neurons, yielding an overall quadratic-like increment in the network mean firing rate as the heterogeneity level increased.

The situation is more complex in the present case, where we have two different and interconnected populations of neurons (the excitatory and the inhibitory population), and also one heterogeneity parameter for each population. The results from the mean-field approach as well as from the numerical simulations can be seen in Figures 2C,D. Figure 2C shows the effect of increasing the excitatory heterogeneity in the activity level of the system. Our mean-field prediction, which agrees very well with numerical simulations, shows that increasing the heterogeneity level of the excitatory population leads to a rise in both excitatory and inhibitory activity. This can be easily understood by considering that the increase of *w_E_* produces the effect in the excitatory firing rate described above, and this in turn increases the input entering from the excitatory to the inhibitory neurons, rising the inhibitory firing rate as well. The increment in the inhibitory rate also modulates back the excitatory population, which implies that the effect of *w_E_* on the excitatory population is not as pronounced as for the case of an isolated excitatory population.

The effects of increasing the inhibitory heterogeneity are, however, qualitatively different from those produced by its excitatory counterpart. As Figure 2D shows, increasing the inhibitory heterogeneity produces a rise in the inhibitory activity but decreases the excitatory activity. The origin of this effect is that increasing *w_I_* leads to the appearance of low-threshold inhibitory neurons with high firing rates, which increases the firing rate of the inhibitory network. This in turn induces more inhibition in the excitatory population, which lowers its level of activity as a consequence. It is interesting to note that, due to the negative character of the feedback within the inhibitory population, the increment in the inhibitory firing rate with the inhibitory heterogeneity is only due to the appearance of low-threshold inhibitory neurons, which pull the average firing rate up. On the other hand, we have three different factors that pull this average down: (i) the appearance of high-threshold inhibitory neurons, (ii) the decay in the positive contribution of the excitatory firing rate, and (iii) the presence of negative feedback within the inhibitory population. Because of this, the increase in the inhibitory firing rate with *w_I_* is not as strong as the increase of the excitatory firing rate with *w_E_*, where the feedback is positive. In particular, the effect of *w_E_* on the excitatory firing rate is about twice as strong as that of *w_I_* in the inhibitory firing rate, as one can see from the differences in the scale of the vertical axis in Figures 2C,D.

### 3.2. Heterogeneity as a gain control mechanism

After observing the strong effect that heterogeneity has on the mean firing rate of a cortical network for a given external input, an immediate question follows: how does neural heterogeneity influence the general input–output properties of cortical circuits? A first approach to answering this question is to analyze the effect of heterogeneity on the input–output dependence, or f-I curve, of the neural network. The f-I curve of a given neural system gives the relationship between a slow (usually considered constant) input to the circuit and the *readout* or mean firing rate of that circuit. There are a number of biophysical mechanisms which are able to modify or control the shape of this curve (a strategy commonly referred to as gain control). One can typically distinguish between several types of gain control, the most common ones being subtractive, divisive, or non-monotonic gain control (see Mejias et al., 2014 for an example of a system able to display these three regimes).

**Figure 3:**
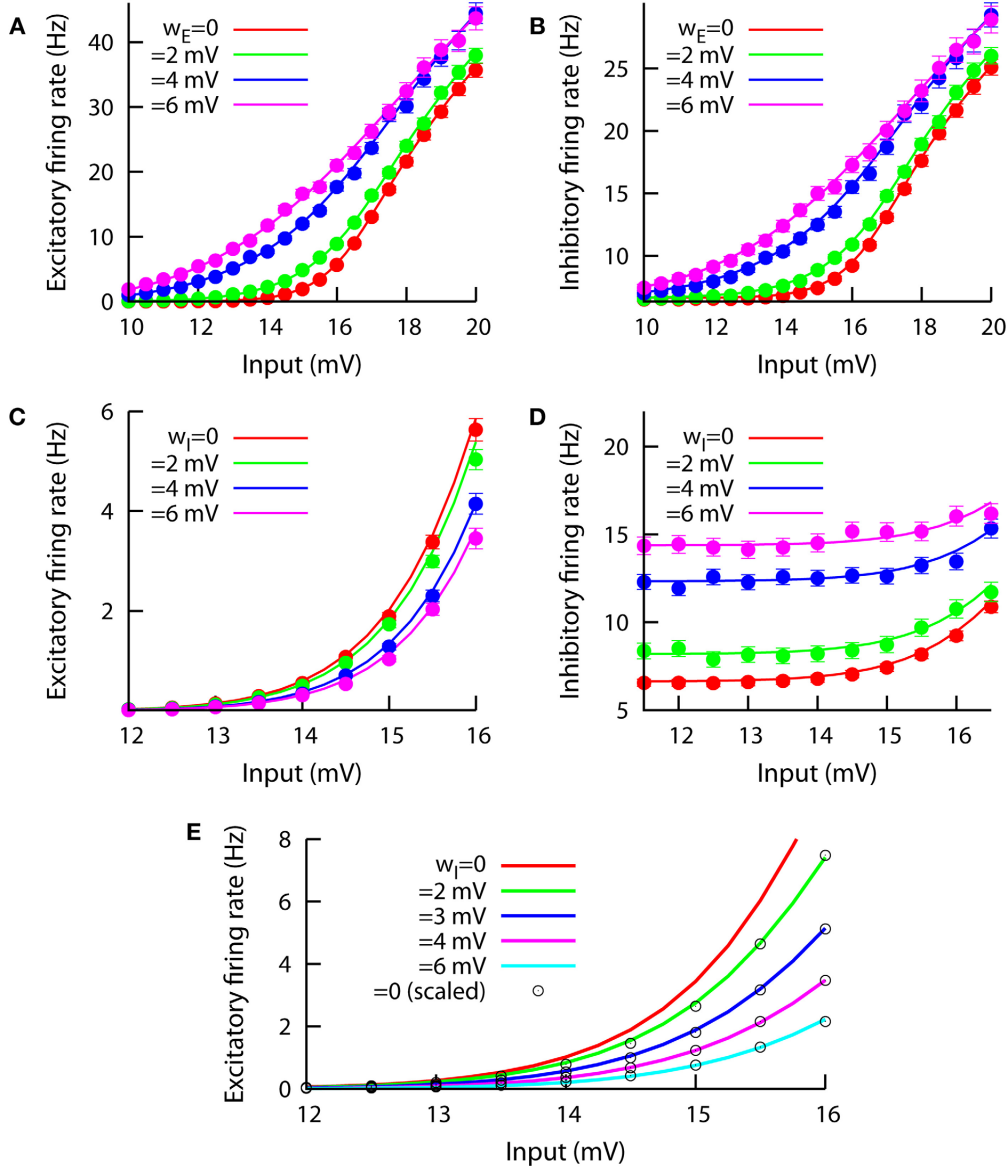
**Effect of modifying *w_E_* (top panels A,B, we keep *w_I_* = 0.1 mV) and *w_I_* (bottom panels C,D, we keep *w_E_* = 0.1 mV) on the f-I curves of the system**. The input in the horizontal axis corresponds to the external excitatory input μ. The inhibitory bias remains at μ_0_ = 17 mV at all times. Left panels show the effect on the excitatory population, while right panels do the same for the inhibitory population. In **(C)**, one can observe a clear divisive gain control of the excitatory f-I curve when *w_I_* varies. This divisive effect is not present in the inhibitory f-I curve, as **(D)** shows. **(E)** The same divisive effect as in **(C)** is displayed, but for μ_0_ = 12 mV and *J_EI_* = −0.4 mV. Circles correspond to the *w_I_* = 0 case (red line) but rescaled by a constant factor to fit the other cases, indicating that the effect of *w_I_* can be described as divisive gain control. The constant factor ζ used to rescale the *w_I_* = 0 f-I curve was obtained for each case by minimization of the squared distance Δ (see main text) and the resulting values are, for increasing *w_I_*, the following: 0.772, 0.531, 0.355, and 0.223.

In this section, we will use our mean-field approach, together with numerical simulations, to address the role of neural heterogeneity as a relevant factor for gain control. Our results are shown in Figure 3, where different f-I curves are plotted, for both the excitatory and the inhibitory populations and varying either *w_E_* or *w_I_*. The effect of the excitatory heterogeneity on the f-I curves of the system is shown in the top panels of the figure, where we can see that increasing *w_E_* linearizes the f-I curve for both the excitatory (Figure 3A) and the inhibitory (Figure 3B) populations. The effect of the excitatory heterogeneity is similar to the noise-induced linearization effect in a recurrent spiking neural network (Sutherland et al., 2009). As in the case of temporal fluctuations in noisy input currents, the effect of cell-to-cell heterogeneity is particularly important around the onset of the f-I curve, when most of the neurons lie between the fluctuation-driven and the mean-driven regime.

The effect of increasing the heterogeneity level in the inhibitory population is, however, more complex. As we can see in Figure 3C, the increase of *w_I_* leads to a decrease in the excitatory firing rate for any given input value. Interestingly, the effect of *w_I_* on the excitatory f-I curve is of a divisive nature, meaning that the inhibitory heterogeneity can be used as a divisive gain control parameter to perform multiplicative and divisive operations in cortical computations. Such a divisive gain control effect holds for small and moderate values of the f-I curve, although as we move to strongly mean-driven conditions (i.e., μ ≥ 20 mV) the divisive control is lost (not shown). The main effect of increasing *w_I_* on the inhibitory population is an increase in the mean firing rate for a wide range of input values, as Figure 3D shows; no clear subtractive, divisive or linearization effect is apparent for the inhibitory cells as their heterogeneity is increased.

Due to the relative complexity of the model (and, in particular, its recurrent connectivity nature), it is not easy to obtain, even approximately, a theoretical proof of the divisive nature of the gain control effect of *w_I_*. Indirect measurements, however, can be used to test this hypothesis. For instance, in Figure 3E we have computed, using our mean-field approach, the f-I curve for the excitatory population and different values of *w_I_*. We have set μ_0_ = 12 mV and *J_EI_* = −0.4 mV so that the divisive effect is stronger and more easily identifiable than in Figure 3C for the range of biases shown. By taking the f-I curve for *w_I_* = 0 (in red) and multiplying it by a given constant factor ζ, one can obtain an f-I curve for *w_I_* > 0. To do this systematically, we have defined the squared distance between the rescaled *w_I_* = 0 f-I curve [namely ζ *r*_0_ (I)] and a given *w_I_* > 0 f-I curve [namely *r_w_(I)*] as

(4) $$\Delta =\frac{1}{n}\sum_{i=1}^{n}\;{\left[\zeta \;r_{0}(i)-r_{w}(i)\right]}^{2},$$

where the subindex *i* runs over all the input values considered in the numerical evaluation of the curve, and *n* = 25 is the total number of these values. By systematically varying the rescaling constant factor ζ, we find the value of this factor that minimizes the squared distance between both curves. This fitting is possible for all values of *w_I_* considered, and the squared distance at the optimal rescaling factor is always small (<0.003). For instance, by multiplying the original (*w_I_* = 0) f-I curve by a factor of ζ = 0.772, we obtain an f-I curve that fits very well (Δ < 0.0022) the f-I curve for *w_I_* = 2 mV. The good overlap between the rescaled *w_I_* = 0 curve (circles in Figure 3E) using different multiplicative constants and the original mean-field solutions (in colored lines), as demonstrated by the small values of Δ obtained and graphically displayed in Figure 3E, indicates that the observed effect is indeed divisive. Simulation results are not displayed for Figure 3E for an easier visual comparison with the rescaled curves, although simulations agree very well with the mean-field predictions as in the previous set of parameters (see Figure 3C for a reference). We have further assessed the goodness of fit by checking that the residuals for each fit are distributed around zero, with approximately two thirds of the data points falling within one standard deviation of the data distribution, as expected for zero-mean Gaussian statistics. Other quantities for measuring the goodness of fit, like a normalized version of the quantity used here (which prevents our fit to depend on the average firing rate), also give the same results.

### 3.3. Stability and phase diagram

**Figure 4:**
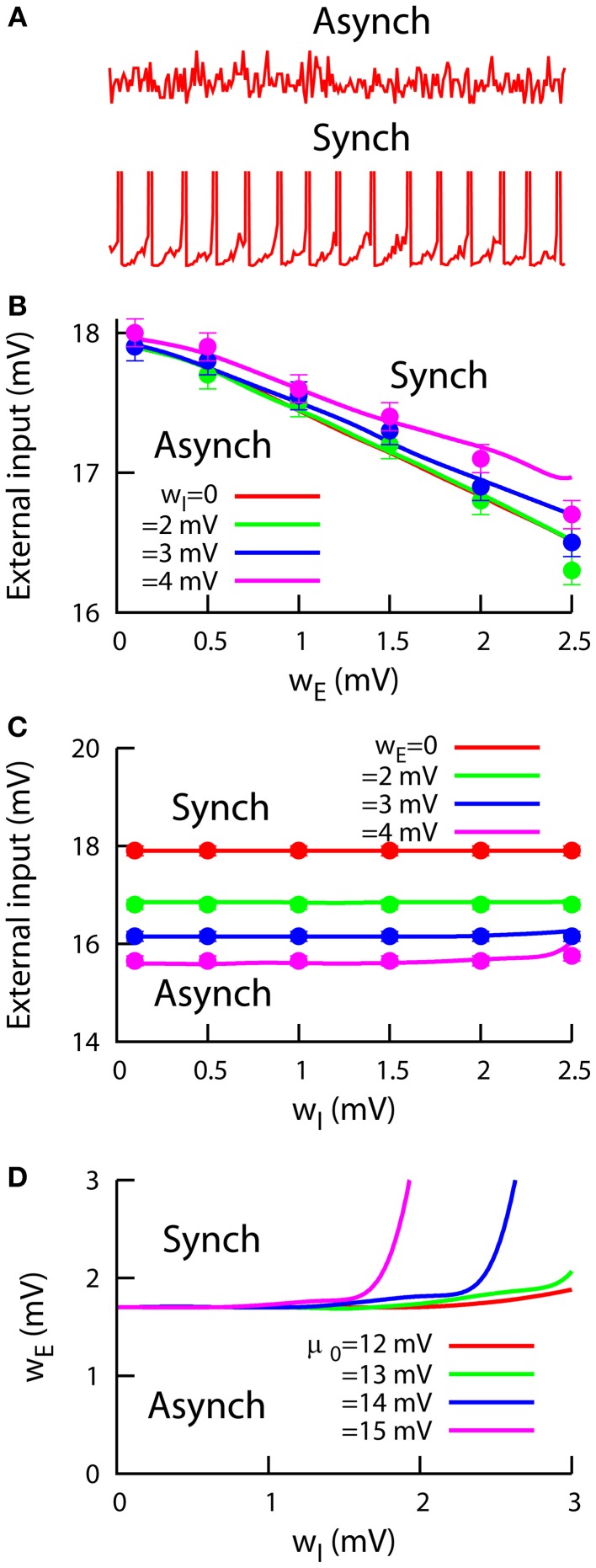
**Stability of networks of heterogeneous neurons. (A)** Examples of the excitatory network mean firing rate in the asynchronous (top) and synchronous (bottom) regimes. **(B)** Critical external input as a function of the excitatory heterogeneity *w_E_*, for μ_0_ = 12 mV and different values of *w_I_*. An external input higher than the critical one will induce spontaneous synchronization in the network. Mean-field predictions (lines) agree with numerical simulations (symbols). **(C)** Critical external input as a function of *w_I_*, for μ_0_ = 12 mV and different values of *w_E_*. **(D)** Phase diagram in the *w_E_* − *w_I_* space, obtained using the mean-field approach from **(B,C)**, for an external input of 17 mV and different levels of the inhibitory population bias μ_0_. For all panels, σ = 1 mV.

So far, we have described the behavior of our cortical network model by assuming fixed point conditions, which led us to asynchronous steady-state solutions of the dynamics. Spiking neural networks are known to display other non-linear dynamics for certain conditions, such as multistability (Compte et al., 2000; Wang, 2001), fast global oscillations (Brunel and Hakim, 1999; Brunel, 2000), or winner-take-all dynamics (Wang, 2002; Wong and Wang, 2006). Although our aim in this study is not to fully characterize this kind of behavior in heterogeneous cortical networks– which would require more advanced calculations– we can study the local stability of the dynamics of the network to map the regions in the parameter space where our conclusions hold. To accomplish this, we compute the Lyapunov exponents of our system (see Equation 15 in Supplementary Material) and estimate, for a given value of the heterogeneity parameters, the maximum external input μ for which the asynchronous steady-state is stable (i.e., all eigenvalues have a negative real part). This limit would give us a clear frontier between asynchronous (top panel in Figure 4A) and synchronous (bottom panel in Figure 4A) network mean firing rate.

Figure 4B shows this maximum external input as a function of the excitatory heterogeneity, for different values of inhibitory heterogeneity. We can observe that, for each *w_I_* value, the maximum external input decreases linearly with the level of excitatory heterogeneity, as in the previously studied case of a purely excitatory heterogeneous network (Mejias and Longtin, 2012). This indicates that networks with highly heterogeneous excitatory neurons are able to enter the synchronous regime with less external stimuli than for the homogeneous case. The observed early synchronization in heterogeneous networks arises due to the presence of low-threshold excitatory neurons. This subset of neurons has a higher firing rate, and therefore they generate a stronger recurrent input that makes them closer to the bifurcation point from asynchrony to synchrony. As a consequence, low-threshold neurons become synchronous with less external input and they in turn contribute to the early synchronization of the rest of the neurons in the network, as we observe in Figure 4B.

The effect of the inhibitory heterogeneity is much less significant (see Figure 4C), although one can distinguish a small increase in the maximum external input for large enough values of *w_I_*. This is to be expected, since a large *w_I_* value would increase the inhibitory firing rate, inducing a decrease in the excitatory firing rate that must be compensated with a higher external input. Therefore, the synchronization onset will be located at a higher external input value. For both panels (Figures 4B,C), numerical simulations (points) agree very well with our mean-field predictions (lines). For large heterogeneity values (*w_E_*, *w_I_* ≥ 2.5 mV), the quenched disorder together with the stochasticity of the system make it difficult to accurately detect the synchronization onset. To avoid this problem, we have restricted our analysis to situations in which both the excitatory and inhibitory heterogeneity levels were small (*w_E_*, *w_I_* < 2.5 mV).

We have also used our mean-field approximation to compute a *w_E_* − *w_I_* phase diagram of the behavior of the system, which is shown in Figure 4D. For both *w_E_* and *w_I_* small, the system is in the asynchronous regime. The asynchronous state continues being stable for increasing *w_I_*, since the subsequent increment in the inhibitory firing rate contributes to stabilize the network dynamics as explained above (see Figure 4C). Only when *w_I_* takes moderate values and *w_E_* is significantly increased, synchronous behavior appears in the network dynamics. As the inhibitory bias μ_0_ takes larger values, the stabilizing effect of increasing *w_I_* reduces the area of the regime of synchronous dynamics. This is due to the fact that *w_I_* allows for a stronger modulation of the inhibitory firing rate when μ_0_ is larger, as the activity of the inhibitory low-threshold neurons will be higher in this case.

It is interesting to highlight that the results presented here (together with other recent studies such as Mejias and Longtin, 2012) provide counter-intuitive situations where heterogeneity promotes synchronization rather than impede it (Borgers and Kopell, 2003; Denker et al., 2004). A comprehensive study of the contrast between our results and the dynamical mechanisms previously reported is, however, beyond the scope of this study and will be addressed in future work.

### 3.4. Signal detection

Since both excitatory and inhibitory heterogeneity have a significant impact on the input–output characteristics for constant input, it is convenient to extend our analysis to consider the effect of heterogeneity in the transmission of more realistic, time-varying signals. In particular, previous work showed that the presence of a certain level of heterogeneity can optimize the transmission of slow signals under rate coding in excitatory populations (Mejias and Longtin, 2012). The phenomenon was also present in cortical-like networks with sparseness and inhibition, although in this more realistic case, no theoretical approximations were provided to support these claims.

**Figure 5:**
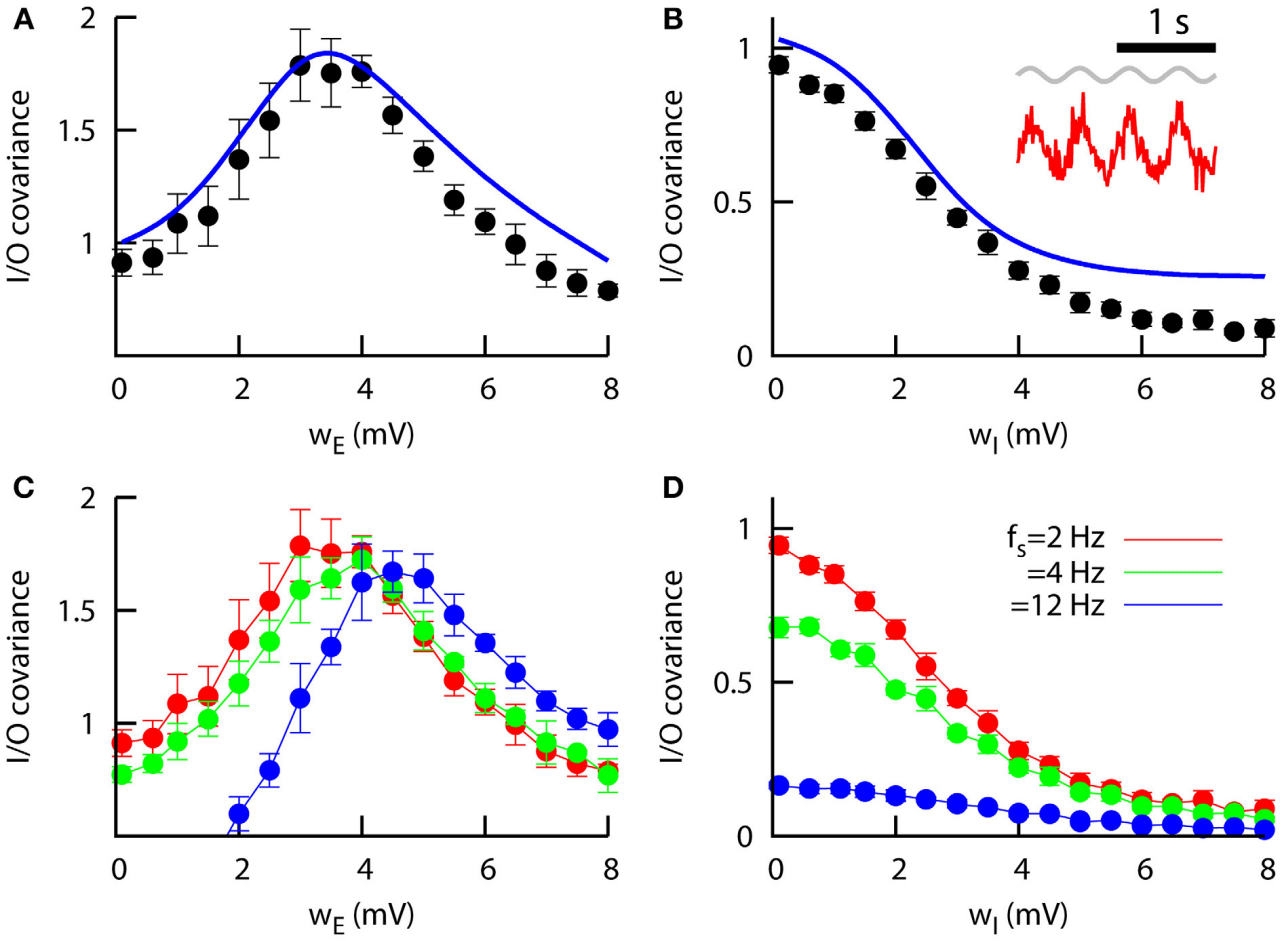
**Signal transmission in networks of heterogeneous neurons. (A)** Zero-lag input–output covariance as a function of the excitatory heterogeneity in the network, for *w_I_* = 0.1 mV. The peak indicates that a specific level of heterogeneity optimizes signal transmission. The mean-field approach (line) reproduces the numerical findings (symbols). Other parameters are *J_EE_* = *J_IE_* = 0.043 mV, *J_EI_* = *J_II_* = −0.06 mV, and μ = μ_0_ = 15 mV, and the signal is characterized by *S*_0_ = 0.5 mV and *f_s_* = 2 Hz. **(B)** Same as in **(A)**, but as a function of the inhibitory heterogeneity and for *w_E_* = 0.1 mV. Inset: example of a slow, weak sinusoidal signal (in gray) driving the excitatory mean firing rate (in red). This situation would correspond to the peak in **(A)** (optimal I/O covariance). **(C)** Same as **(A)**, but for different input frequencies. Only numerical results (averaged over 15 trials) are presented in this case, since the mean-field predictions does not hold for high input frequencies. **(D)** Same as **(B)**, but for different input frequencies and only considering the numerical results. **(C,D)** share the same color code.

The mean-field approximation presented in the Supplementary Material (see Section Mean-field approach) constitutes a useful tool to investigate these heterogeneity-induced resonances in cortical-like network models, and to evaluate the likelihood of this phenomenon to occur in real cortical circuits. We consider an external input constituted by

(5) $$\hat{\mu }(t)=\mu +S_{0}\sin(2\pi f_{s}t),$$

with the first part of the r.h.s. being a constant input and the second part being a slow and weak modulation. Such a weak input modulation is able to drive the excitatory mean firing rate of the network under certain circumstances, a situation which is shown in the inset of Figure 5B. A convenient measurement to quantify this behavior is the zero-lag input–output covariance function, which is given by

(6) $$C\equiv \langle \hat{\mu }(t)\nu_{E}(t)\rangle -\langle \hat{\mu }(t)\rangle \langle \nu_{E}(t)\rangle .$$

Figure 5A shows this input–output covariance as a function of the excitatory heterogeneity. For networks of homogeneous neurons, the modulation part of the signal is typically too weak to be noticed. For higher values of the excitatory heterogeneity, however, the sensitivity of the network to small inputs increases, due to the existence of a larger number of low-threshold excitatory neurons. As a consequence, the small input modulation is now able to strongly drive the output (i.e., the excitatory network mean firing rate). For even higher values of *w_E_* the overall activity of the network increases drastically and the variations due to input modulations become slightly weaker. This makes the signal-driven firing rate modulations too small compared to the baseline firing rate, and as a consequence the quality of signal transmission to a given linear readout system decreases. The overall effect is a bell-shape dependence of the input–output covariance with *w_E_* shown in Figure 5A, indicating that a certain non-zero level of excitatory heterogeneity optimizes signal transmission, as in the simpler case of a purely excitatory population studied in Mejias and Longtin (2012). Furthermore, our novel mean-field approach for excitatory and inhibitory sparse populations closely follows the numerical results.

The effect of inhibitory heterogeneity on signal transmission is notably different from the situation explained above. As we can see from Figure 5B, the input–output covariance tends to be weaker in networks whose inhibitory neurons are highly heterogeneous. This is due to the fact that inhibitory heterogeneity causes an increase in the inhibitory firing rate, which reduces the sensitivity of the excitatory population to weak stimuli and therefore hinders its capacity for signal detection. This is consistent with recent experimental and theoretical findings which show that correlations between two neurons decrease as their firing rate decrease (de la Rocha et al., 2007), and it suggests that heterogeneity in inhibitory neurons may have an important role in decorrelation between input signals and neural activity.

We can also see that the detection of the signal is frequency-dependent. In Figure 5C the input–output covariance as a function of *w_E_* is computed for different values of the signal frequency *f_s_*, with a small shift of the peak toward more heterogeneous networks as the input frequency is increased. This behavior was also observed for the case of one isolated excitatory population (Mejias and Longtin, 2012), and suggests that the ability of neural networks to efficiently detect and transmit signals of a given frequency range depends on the heterogeneity level of the network. The decrease of the signal detection as a function of the inhibitory heterogeneity also depends on the input frequency considered (Figure 5D), although for large enough *w_I_* the signal is not detected regardless of the frequency.

### 3.5. Multiple heterogeneity sources

As a final remark, it should be noted that, in all of the simulations and analyses presented so far, either the excitatory or the inhibitory heterogeneity was varied, while the heterogeneity of the other population was kept fixed at a very low level (0.1 mV, which would correspond to a almost homogeneous population). In the situations in which the system under study behaves in a linear fashion and the effects caused by parameter variations are independent, this approach is convenient to systematically characterize the behavior of the system. The neural network under study, however, is known to display multiple kinds of non-linear behavior (such as, for instance, the non-linear dependence of the mean firing rate with the heterogeneity level, as shown in Figures 2C,D). It is, therefore, unclear whether one can infer the response of the system for arbitrary combinations of heterogeneity parameters from the curves and results presented in previous sections, in which mainly only one type of heterogeneity was analyzed at a time.

**Figure 6:**
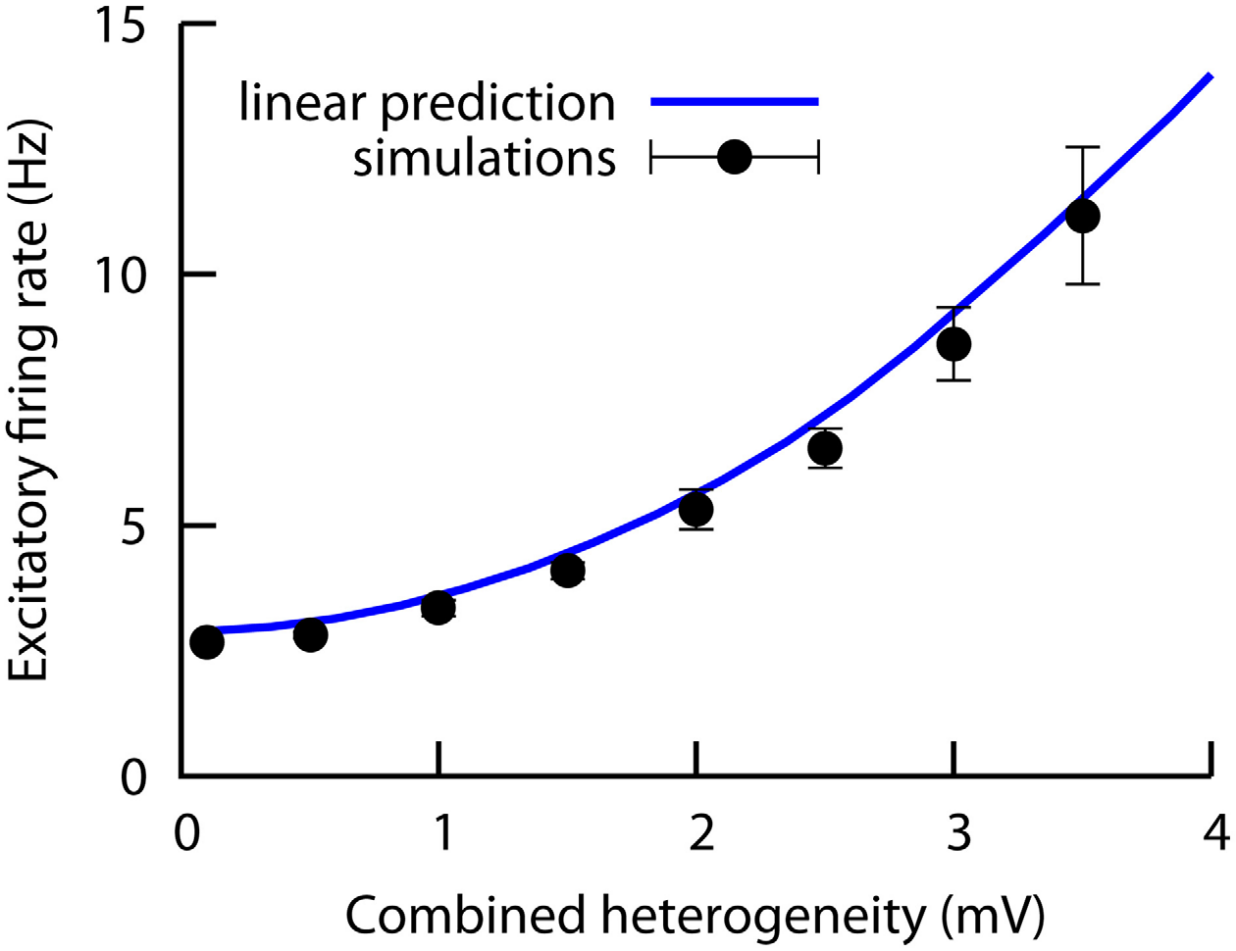
**Numerical results (symbols) of the excitatory firing rate for a network in which both the excitatory and the inhibitory heterogeneity levels are increased simultaneously (i.e., we plot the excitatory firing rate as a function of the combined heterogeneity, defined as *W* = *w_E_* = *w_I_*)**. The solid line is obtained with the mean-field curves of Figures 2C,D (red curves), and assuming that both contributions add up in a linear fashion. The agreement between simulations and the mean-field under the linear hypothesis indicates that the system behaves linearly in this case. All parameters as in Figures 2C,D.

In order to test the validity of our results in more complex scenarios, we have jointly increased both heterogeneity levels (*w_E_* and *w_I_*) at the same time, and we numerically computed the excitatory mean firing rate of the network as a function of this *combined* heterogeneity level *W* (with *W* = *w_E_* = *w_I_*). To test the linearity of the system to the presence of multiple sources of heterogeneity, we also compute, using the mean-field solution, the changes in the excitatory firing rate due to only *w_E_* or only *w_I_* (while keeping the other heterogeneity level at zero value), and we add these two contributions together. The comparison is shown in Figure 6, where we can see that the simulation results closely follows the linear prediction up to values of the combined heterogeneity of ~4 mV. This finding implies that the effects of multiple types of heterogeneity can add up linearly in some parameter regimes, such as the one in this preliminary investigation. Therefore, the results of the present work are also valid for more realistic situations in which different types of heterogeneity (i.e., excitatory and inhibitory) are simultaneously present in the system.

## 4. Discussion

The importance and roles of intrinsic neuronal heterogeneities on the dynamics of neural networks is starting to being uncovered in recent years (Marsat and Maler, 2010; Padmanabhan and Urban, 2010; Savard et al., 2011; Angelo et al., 2012; Tripathy et al., 2013). Although commonly disregarded in most modeling studies, an increasing level of attention has been drawn to the subject by theoretical and computational models as well (Golomb et al., 2001; Denker et al., 2004; Luccioli and Politi, 2010; Mejias and Longtin, 2012; Nicola and Campbell, 2013). In particular, novel theoretical frameworks for addressing the heterogeneity of neural systems have been proposed recently. Yim and colleagues, for example, propose a theoretical approach especially useful for addressing the relationship between neural heterogeneity and neural correlations (Yim et al., 2013), and they sketch a possible explanation for recent evidence of a positive role of heterogeneity on population coding (Padmanabhan and Urban, 2010). In another recent work, Nicola and Campbell provide a set of mean-field approaches used to shed light onto a heterogeneity-induced change on the nature of the Hopf bifurcation responsible for burst generation (Nicola and Campbell, 2013).

The theoretical understanding of the effects of heterogeneity on neural systems is still a young problem, though, and only simple situations have been considered up to now. In this work, we have analytically and computationally studied the interplay between population-specific levels of cellular heterogeneity, an important problem that has not been properly addressed to date. Interestingly, the effects that excitatory heterogeneity produces on neural networks are quite different from the ones produced by inhibitory heterogeneity. Excitatory heterogeneity, as we have shown, non-linearly increases the network mean firing rate with respect to that of a homogeneous network, and the f-I curves of the system are linearized as well. In this sense, excitatory heterogeneity may be viewed as a classical *quenched disorder* in excitable systems, with similar effects on the f-I curve than that of pure noise (Doiron et al., 2001). On the other hand, the introduction of inhibitory heterogeneity induces an increase (with respect to homogeneous networks) in the inhibitory firing rate and a decrease in the excitatory firing rate, and a divisive modulation of the f-I curve as a result. Divisive gain control mechanisms is often assumed as a key operation for neural computations (Carandini and Heeger, 1994; Chance and Abbott, 2000), but biophysical mechanisms for such a modulation have been hard to identify, regardless of being network-based mechanisms (Holt and Koch, 1997; Doiron et al., 2001; Chance et al., 2002; Mejias et al., 2014) or cell-based mechanisms (Prescott and De Koninck, 2003; Mehaffey et al., 2005). The identification of neuronal heterogeneity in inhibitory populations as a biophysically realistic mechanism for multiplicative and divisive gain control constitutes one of the key achievements of the present study.

The analysis of the stability of the fixed point solutions of heterogeneous networks also provides useful information about the effects of heterogeneity on neural networks. Again, the effects of neural heterogeneity heavily depend on the population in which it is found. Excitatory heterogeneity leads to an easier spontaneous synchronization of the neural network, while inhibitory heterogeneity has a weaker effect and tends to slightly increase the robustness of the asynchronous state. This produces a rich repertoire of stability behaviors in neural networks, with the stability conditions of a given particular network depending on its balance between excitatory and inhibitory heterogeneity.

In recent works, an optimal information transmission has been shown to occur for heterogeneous populations of neurons (Marsat and Maler, 2010; Padmanabhan and Urban, 2010), and the presence of short-term synaptic plasticity has been suggested to increase the efficiency of coincidence detection in the presence of heterogeneity via the appearance of *optimal frequencies* (Mejias and Torres, 2008). These findings indicate that heterogeneity may have an important role in information transmission in neural systems. In the present study, we have demonstrated here that the optimization of signal detection by networks of heterogeneous neurons under rate coding, first described in Mejias and Longtin (2012), holds for the more realistic cortical-like network used here, by means of both numerical simulations and mean-field approaches. The improvement of signal detection in heterogeneous neural and excitable systems has been a recent focus of interest. For instance, Tessone et al. (2006) found that global synchronized events in response to weak, slowly modulated external signals can be optimized in heterogeneous networks, a result that has been also obtained in neural networks with electrical and chemical synapses (Perez et al., 2010). Global synchronized events in heterogeneous networks can also work at very short time scales, being triggered by fast input and allowing for an efficient temporal coding (Mejias and Longtin, 2012). Recent experimental work has also highlighted an optimization of population coding in networks of heterogeneous neurons (Marsat and Maler, 2010; Padmanabhan and Urban, 2010; Savard et al., 2011; Angelo et al., 2012), establishing a solid ground for neural heterogeneity as a key ingredient of neural coding.

Finally, it is worth noting that, although we have studied exclusively the case of heterogeneity in the distance-to-threshold of LIF neurons, our mean-field approach can be used to study heterogeneity in other parameters as well. Indeed, there are many potential biophysical sources of heterogeneity in neural systems, both at the network level (i.e., heterogeneity in the network connectivity, as in Olmi et al., 2010) and at the neuron level. In this second group, possible heterogeneity sources can be defined in terms of anatomical and morphological properties, or also at a functional level, including neuronal excitability (Tessone et al., 2006; Perez et al., 2010), different degrees of spike frequency adaptation (Hemond et al., 2008; Nicola and Campbell, 2013), or other biophysical properties (Padmanabhan and Urban, 2010; Tripathy et al., 2013), to name a few. Understanding their individual or joint role in neural dynamics will require future modeling work at different scales and levels of detail, for which mean-field approaches could be of great help.

### Conflict of interest statement

The authors declare that the research was conducted in the absence of any commercial or financial relationships that could be construed as a potential conflict of interest.
