# Supplementary material for: Differential effects of excitatory and inhibitory heterogeneity on the gain and asynchronous state of sparse cortical networks
Source: Front Comput Neurosci. 2014 Sep 12;8:107. doi: 10.3389/fncom.2014.00107 (PMC4162374; doi:10.3389/fncom.2014.00107)
Supplement: Supplementary file 1 [file Presentation1.PDF]

Appendix: Differential effects of excitatory and inhibitory heterogeneity on the gain and asynchronous state of sparse cortical networks

Jorge F. Mejias and André Longtin

August 27, 2014

1 Mean-field approach

Considering the diffusion approximation in the neural input as in (**Brunel, 2000**), the dynamics of excitatory and inhibitory neurons may be written, respectively, as

$$\tau_m \frac{dV_i(t)}{dt} = -V_i(t) + \mu_E(t) + \sigma_E(t)\sqrt{\tau_m}\xi_i(t), \tag{1}$$

$$\tau_m \frac{dV_i(t)}{dt} = -V_i(t) + \mu_I(t) + \sigma_I(t)\sqrt{\tau_m}\xi_i(t), \tag{2}$$

with the terms of the input current given by

$$\begin{aligned} \mu_E(t) &= \mu + \tau_m K\gamma J_{EE}\nu_E(t) + \tau_m K(1-\gamma)J_{EI}\nu_I(t), \\ [\sigma_E(t)]^2 &= \sigma^2 + \tau_m K\gamma J_{EE}^2\nu_E(t) + \tau_m K(1-\gamma)J_{EI}^2\nu_I(t), \end{aligned} \tag{3}$$

$$\begin{aligned} \mu_I(t) &= \mu_0 + \tau_m K\gamma J_{IE}\nu_E(t) + \tau_m K(1-\gamma)J_{II}\nu_I(t), \\ [\sigma_I(t)]^2 &= \sigma^2 + \tau_m K\gamma J_{IE}^2\nu_E(t) + \tau_m K(1-\gamma)J_{II}^2\nu_I(t). \end{aligned} \tag{4}$$

Here, $\nu_E(t), \nu_I(t)$ are the instantaneous network mean firing rate of the excitatory and inhibitory populations, respectively, assuming that the network is in an asynchronous activity state.

One may compute the mean firing rate of the i-th neuron, namely $f_{\alpha,i}$ (where α is the population label), by using the first-passage time calculation for a leaky IF neuron model (see **Tuckwell, 1989**). This yields

$$f_{\alpha,i} = \left[\tau_{ref} + \tau_m \int_{y_r^{\alpha,i}}^{y_\theta^{\alpha,i}} F(z)dz\right]^{-1}, \tag{5}$$

where $y_\theta^{\alpha,i} = \frac{\theta_{\alpha,i}-\mu_\alpha}{\sigma_\alpha}$, $y_r^\alpha = \frac{V_r-\mu_\alpha}{\sigma_\alpha}$ and $F(z) = \sqrt{\pi}\exp(z^2)(1+\mathrm{erf(z)})$. In order to obtain the stationary mean firing rate of each population, namely ν_E, ν_I, we have to average over the stationary single-neuron rates $f_{E,i}$ and $f_{I,i}$, respectively. If the network is large enough ($N \to \infty$), it is a good approximation to substitute the sum over the neuron firing rates by an integral over the neuron

firing rates, given a probability distribution of rates $P_E(f_E)$ for the excitatory population and $P_I(f_I)$ for the inhibitory population, that is,

$$\nu_\alpha = \frac{1}{N_\alpha}\sum_{i=1}^{N_\alpha} f_{\alpha,i} \simeq \int_0^{1/\tau_{ref}} f_\alpha \; P_\alpha(f_\alpha) df_\alpha \tag{6}$$

where N_α is the number of neurons in the population α (that is, γN for excitatory population, and $(1-\gamma)N$ for inhibitory population). We assume now that the variability in the neuron firing rates is caused by the heterogeneity of firing threshold values, which is characterized by the Gaussian distribution $P_\alpha(\theta_\alpha)$ for the population α. It is worth noting that, for large enough networks, it is the firing threshold variability across a given population α what causes the individual firing rate variability across that same population α. Threshold variability across inhibitory neurons, for instance, has approximately the same impact on every excitatory neuron (since any given excitatory neuron integrates input coming from a large number of inhibitory neurons), and therefore it could not be responsible for individual firing rate differences across excitatory neurons.

Assuming this relationship between the firing rate variability and the threshold variability, one can consider $P_\alpha(f_\alpha)df_\alpha = P_\alpha(\theta_\alpha)d\theta_\alpha$ and transform the previous integral over rates in an integral over thresholds. This lead us to the expression for the stationary states ν_E, ν_I of a network of heterogeneous neurons (where, as usual, $\alpha = \{E, I\}$):

$$\nu_\alpha = \int_{\theta^\alpha_{min}}^{\theta^\alpha_{max}} \left[\tau_{ref} + \tau_m \int_{y^\alpha_r}^{y^\alpha_\theta} F(z)dz\right]^{-1} P_\alpha(\theta_\alpha)d\theta_\alpha, \tag{7}$$

where $y^\alpha_\theta = \frac{\theta_\alpha - \mu_\alpha}{\sigma_\alpha}$, and θ^α_{min}, θ^α_{max} are the integration intervals. These intervals, in practice, have to be conveniently chosen. Concretely, they must satisfy $\theta^\alpha_{max} \gg \overline{\theta} + w_\alpha$, and $V_r \ll \theta^\alpha_{min} \ll \overline{\theta} - w_\alpha$. These restrictions impose a range of validity of w_α for our theory, however this range is wide enough to include the values observed in experiments, where the dispersion in neural thresholds is around a few millivolts.

Let us define the right-hand side expression of Eq. (7) as $\Phi_\alpha(\nu_E, \nu_I, w_E, w_I)$. We may analyze the stability of the fixed point solutions by employing a simplified treatment of the firing rate dynamics of our system. Concretely, for asynchronous activity states, we may assume that the dynamics of the network firing rate evolves according to

$$\tau_\nu \, \frac{d\nu_\alpha}{dt} = -\nu_\alpha + \Phi_\alpha(\nu_E, \nu_I, w_E, w_I), \tag{8}$$

where $\tau_\nu \simeq 3\ ms$ is the typical time scale of the rate dynamics (for further analysis, see **Gerstner, 2000**). For this case, we can employ standard techniques to study the linear stability of Eq. (8). Concretely, we linearize Eq. (8) around the fixed point solution, and we then compute the eigenvalues of the Jacobian matrix associated with the linearized system. After some algebra, we obtain a set of eigenvalues $\{\lambda_+, \lambda_-\}$ given by

$$\lambda_\pm = \frac{Z_A + Z_D}{2} \pm \frac{1}{2}\sqrt{(Z_A + Z_D)^2 - 4(Z_A Z_D - Z_B Z_C)}, \tag{9}$$

where the terms $Z_A,\ Z_B,\ Z_C,\ Z_D$ are

$$Z_A = -1 + \tau_m \int_{\theta_{min}^E}^{\theta_{max}^E} \frac{Y_A(\theta_E)F(y_\theta^E) - Y_A(V_r)F(y_r^E)}{\left[\tau_{ref} + \tau_m \int_{y_r^E}^{y_\theta^E} F(z)dz\right]^2} P_E(\theta_E)d\theta_E,$$

$$Z_B = \tau_m \int_{\theta_{min}^E}^{\theta_{max}^E} \frac{Y_B(\theta_E)F(y_\theta^E) - Y_B(V_r)F(y_r^E)}{\left[\tau_{ref} + \tau_m \int_{y_r^E}^{y_\theta^E} F(z)dz\right]^2} P_E(\theta_E)d\theta_E,$$

$$Z_C = \tau_m \int_{\theta_{min}^I}^{\theta_{max}^I} \frac{Y_C(\theta_I)F(y_\theta^I) - Y_C(V_r)F(y_r^I)}{\left[\tau_{ref} + \tau_m \int_{y_r^I}^{y_\theta^I} F(z)dz\right]^2} P_I(\theta_I)d\theta_I, \tag{10}$$

$$Z_D = -1 + \tau_m \int_{\theta_{min}^I}^{\theta_{max}^I} \frac{Y_D(\theta_I)F(y_\theta^I) - Y_D(V_r)F(y_r^I)}{\left[\tau_{ref} + \tau_m \int_{y_r^I}^{y_\theta^I} F(z)dz\right]^2} P_I(\theta_I)d\theta_I,$$

and the functions $Y_A,\ Y_B,\ Y_C,\ Y_D$ are defined as follows

$$Y_A(x) = \frac{2\tau_m K\gamma J_{EE}\sigma_E^2 + \tau_m K\gamma J_{EE}^2(x-\mu_E)}{\sigma_E^3},$$

$$Y_B(x) = \frac{2\tau_m K(1-\gamma)J_{EI}\sigma_E^2 + \tau_m K(1-\gamma)J_{EI}^2(x-\mu_E)}{\sigma_E^3},$$

$$Y_C(x) = \frac{2\tau_m K\gamma J_{IE}\sigma_I^2 + \tau_m K\gamma J_{IE}^2(x-\mu_I)}{\sigma_I^3}, \tag{11}$$

$$Y_D(x) = \frac{2\tau_m K(1-\gamma)J_{II}\sigma_I^2 + \tau_m K(1-\gamma)J_{II}^2(x-\mu_I)}{\sigma_I^3}.$$

The fixed point solution will be stable as long as both λ_+ and λ_- have a negative real part.

References

Brunel, N. (2000), Dynamics of sparsely connected networks of excitatory and inhibitory spiking neurons, *J. Comp. Neurosci.*, 8, 183–208.

Tuckwell, H. C. (1989), Introduction to theoretical neurobiology. Volume 2: nonlinear and stochastic theories (Cambridge).

Gerstner, W. (2000), Population dynamics of spiking networks: fast transients, asynchronous states, and locking, *Neural Comput.*, 12, 43–89.
